# Maternal and infant microbiome: next-generation indicators and targets for intergenerational health and nutrition care

**DOI:** 10.1093/procel/pwad029

**Published:** 2023-05-15

**Authors:** Shengtao Gao, Jinfeng Wang

**Affiliations:** College of Food Science & Nutritional Engineering, China Agricultural University, Beijing 100083, China; College of Food Science & Nutritional Engineering, China Agricultural University, Beijing 100083, China

**Keywords:** newborn, microbiome, disease detection, pregnancy management, health care products

## Abstract

Microbes are commonly sensitive to shifts in the physiological and pathological state of their hosts, including mothers and babies. From this perspective, the microbiome may be a good indicator for diseases during pregnancy and has the potential to be used for perinatal health monitoring. This is embodied in the application of microbiome from multi body sites for auxiliary diagnosis, early prediction, prolonged monitoring, and retrospective diagnosis of pregnancy and infant complications, as well as nutrition management and health products developments of mothers and babies. Here we summarized the progress in these areas and explained that the microbiome of different body sites is sensitive to different diseases and their microbial biomarkers may overlap between each other, thus we need to make a diagnosis prudently for those diseases. Based on the microbiome variances and additional anthropometric and physical data, individualized responses of mothers and neonates to meals and probiotics/prebiotics were predictable, which is of importance for precise nutrition and probiotics/prebiotics managements and developments. Although a great deal of encouraging performance was manifested in previous studies, the efficacy could be further improved by combining multi-aspect data such as multi-omics and time series analysis in the future. This review reconceptualizes maternal and infant health from a microbiome perspective, and the knowledge in it may inspire the development of new options for the prevention and treatment of adverse pregnancy outcomes and bring a leap forward in perinatal health care.

## Introduction

According to the theory of Developmental Origin of Health and Diseases (DOHaD), the risk of developing noncommunicable diseases like cardiovascular disease, cancer, and chronic respiratory disease in adulthood may be influenced by the exposures and nutrition in the beginning stages of life including fetus, infant, and childhood (Hanson and Gluckman, 2014; Hoffman et al., 2021). Maintaining the health of pregnancy and newborns is of great importance to promote long-term health or decrease the risk of future diseases. However, due to the complex physiological state of pregnancy, timely, and accurate assessment of the health status of the pregnancy and the newborn is not an easy task. The original clinical diagnostic techniques are usually beyond the reach of timeliness and operability. Complementary methods are needed to fill in the gaps.

The human body provides stable and nutrient-rich habitat for a variety of commensal, opportunistic, and pathogenic microorganisms, which together form an ecosystem that is regularly in dynamic balance. The imbalance of such ecosystem is frequently associated with various diseases (Cho and Blaser, 2012), thus microbiome from different body sites have been used to characterize diseases *in situ* or across sites, such as oral microbiome for dental caries and pancreatic cancer (Teng et al., 2015; Fan et al., 2018; Blostein et al., 2022), gut microbiome for nonalcoholic fatty liver disease (Leung et al., 2022) and urinary microbiome for urologic cancers (Whiteside et al., 2015; Aragón et al., 2018). Similarly, the association between microbiome and pregnancy complications has also been extensively studied (Beckers and Sones, 2020; Huang et al., 2021; Zhang et al., 2022; Pinto et al., 2023). Several studies have suggested the great potential of the microbiome in predicting and diagnosing pregnancy complications (Jin et al., 2022; Pinto et al., 2023).

Additionally, the microbiome has the potential for health monitoring during pregnancy and infant growth. For instance, prediction of personalized postprandial glycemic response to real-life meals by machine-learning algorithms integrating blood parameters, dietary habits, anthropometrics, physical activity, and gut microbiome showed a significant association with measured results (Zeevi et al., 2015). Among the above factors, individual-specific microbiome factors contributed predominantly to the considerably postprandial variability glycemic responses to identical meals. Based on this predicted postprandial glycemic response, personalized intervention strategies can be developed for pregnant women with dysbiosis metabolism, especially for women who suffered from gestational diabetes mellitus (GDM). For newborns and infants, the microbiome is being used to measure their physiological development as well as their nutritional status (Smith et al., 2013; Henrick et al., 2021). With the involvement of microbiome information, precise nutritional and dietary management, over-the-counter probiotics/prebiotics may offer better options for mothers and infants who need medical interventions (Chen et al., 2019; Lopez-Moreno and Aguilera, 2020; Samara et al., 2022). A typical case is the usage of *Bifidobacterium* and its prebiotics human milk oligosaccharides (HMOs) to silence intestinal inflammation early in life by dampening inflammatory responses, in particular T helper 2 (Th2)- and Th17-type responses (Henrick et al., 2021). Maternal and neonatal microbiome play an important role in the discovering, safety evaluation, and precision application.

**Figure 1.**
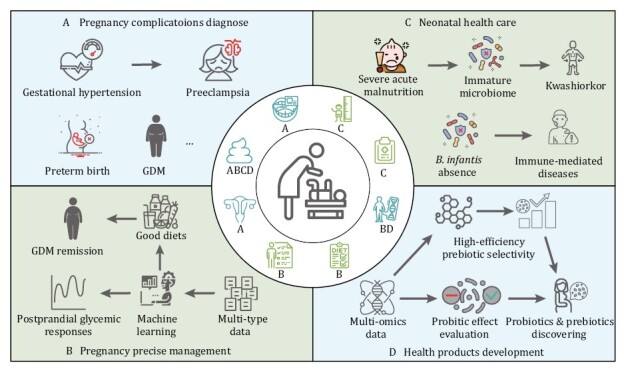
**Overview of maternal and neonatal microbiome utility in perinatal health care.** (A) Microbiome in oral, stool, and vagina are instructive for multiple pregnancy-complications diagnose, prediction, and monitoring. Combining of microbiome in multi body site could efficiently contribute to the early detection of pregnant diseases. (B) Prevention of fetal overgrowth and control of maternal hyperglycemia are the primary goal for GDM treatment, which is usually achieved by dietary modification and promotion of physical activity to minimize postprandial glucose elevations. Encouragingly, machine-learning model trained based on maternal microbiome, meal composition, and genetics data could accurately predict postprandial glycemic responses, which may lay the foundation for precise pregnancy management. (C) The infant microbiome can be used as an indicator and target of flora maturity and nutritional health status, supporting normal immune development and reducing the risk of immune-mediated disease. (D) Multi-omics including microbiome contributes to the prebiotics and probiotics discovering and safety evaluation. The icons in the central loop represent the main data types that used for the relevant sections of maternal and neonatal health care. The markers under each icon represent the application scenario of this type of data. For pregnancy complications diagnose, the datasets including microbiome from oral, stool, and vagina; for pregnancy precise management, the datasets including stool microbiome, physical activity, host genetics, and diets compositions; for neonatal health care, the datasets including stool microbiome, body length, and clinical records data; for health products development, the datasets including stool microbiome and host genetics.

Collectively, recent studies demonstrated a wide usage of microbiome for the maintenance of maternal and neonatal health, thus a systemic summary of the role of microbiome in the intergenerational health and nutrition care is required. This review thus summarized the latest research on maternal and infant microbiomes to map the future of microbiomes in the diagnosis of pregnancy disorders and maternal and neonatal health care (Fig. 1). We searched for studies associated with microbiome of maternal and neonatal health and nutrition care up to March 2023, using the PubMed and the Web of Science databases. The search terms included “pregnancy complication,” “microbiome,” “precise nutrition,” “infant development,” “infant health,” etc. The knowledge in it lays the foundation for using the microbiome as an indicator and target to establish individualized diagnosis and treatment methods, develop surveillance protocols, and guide health care product development. Such fresh perspective to improve the prevention and treatment of maternal and infant-related diseases and to better maintain perinatal health status may benefit mothers and infants suffering from pregnancy complications and adverse pregnancy outcomes, as well as those with a special interest in early-life health.

## Early detection and monitoring of pregnancy complications and adverse pregnancy outcomes with the microbiome

In recent years, because of the complex interplay between demographic and lifestyle factors and delaying childbearing, pregnant women are more likely to have acquired medical disorders. Wherein, GDM, gestational hypertension, preterm birth (PTB), and preeclampsia are the most common complications of pregnancy, with approximate prevalence of 14.80%, 11.95%, 7%, and 4.08% in China, respectively (Juan and Yang, 2020; Li et al., 2020; Yang et al., 2021). In numerous cases, the women occurring pregnancy complications like pregnancy-induced hypertension, preeclampsia, and GDM have no obvious symptoms before or early pregnancy. The diagnoses of these pregnant diseases constantly depend on the parameters that tested after 20 weeks of pregnancy (Sibai, 2003; Weinert, 2010), but there is not yet a cure or an efficacious prevention strategy for those pregnant diseases when it came to late pregnancy (Plows et al., 2018). Actually, many complications are preventable if these pregnant diseases could be detected earlier and managed appropriately by nutrition, exercise, and drug intervention along with heightened monitoring during labor and delivery (Lende and Rijhsinghani, 2020). Short- and long-term risks to mother and newborn due to pregnancy complications can be reduced by early detection or prediction.

### Early prediction

GDM is the most common metabolic disease first seizure during pregnancy (Johns et al., 2018). Its prevalence in some countries or areas is even higher than 20% (Damm and Mathiesen, 2015). Oral glucose tolerance test (OGTT) performed between 24 and 28 weeks of gestation is the gold standard for GDM screening (Olabi and Bhopal, 2009). According to the diagnostic guideline of International Association of Diabetes and Pregnancy Study Groups (IADPSG), fasting plasma glucose ≥5.1 mmol/L or 1-h blood glucose ≥10.0 mmol/L or 2-h blood glucose ≥8.5 mmol/L after 75 g OGTT are the diagnostic thresholds for GDM (IADPSG, 2010). However, OGTT is uncomfortable for some pregnant women and is not tested until a specific gestational week. Early prediction of GDM and avoidance of OGTT are of greatly required in pregnancy, which provides a best scenario for microbiome.

A recent study illustrated an elevated proinflammatory cytokines, decreased fecal short-chain fatty acids (SCFAs), and altered microbiome (mainly decreased *Prevotella copri*, *Lactobacillus ruminis*, and *Actinomyces*) in first trimester samples of women who later developed GDM (Pinto et al., 2023). Then using fecal microbiota transplant (FMT) confirmed that gut microbiome of GDM triggered an inflammation more than 10 weeks before the diagnosis of GDM, which subsequently induced the development of insulin resistance and progression to GDM (Pinto et al., 2023). Following these observations, a Xgboost model combining the first trimester data of microbiome, cytokine profile, and medical history was developed which accurately predicted GDM with prediction accuracy reached 0.83 (auROC, area under the receiver operating characteristic curve) (Pinto et al., 2023). Fecal microbiome features in the first trimester alone result in the accuracy of a GDM prediction model to 0.73 (Pinto et al., 2023), implying a great potential of gut microbiome for GDM prediction.

Another application scenario with great predictive value is PTB. Numerous findings pointed out that intrauterine infection is one of strong risk factors for PTB, where the load and diversity of bacteria rising from the vagina were supposed to contribute to preterm delivery (Hillier et al., 1988, 1993; Romero et al., 1989; Yoon et al., 2001; Gardella et al., 2004; DiGiulio et al., 2008). Consistent with this hypothesis, Fettweis et al. (2019) reported a significant decrease in *Lactobacillus crispatus* and increase in BV-associated bacterium including *Sneathia amnii*, TM7-H1, *Prevotella* species, and nine other taxa in vagina of women who delivered preterm. Further analysis demonstrated that cytokine levels such as interleukin-1β (IL-1β), IL-6, IL-8, eotaxin, tumor necrosis factor-α (TNF-α), IL-17A, major intrinsic protein of lens fiber-1β (MIP-1β), C-X-C motif chemokine ligand 10 (CXCL10), C-C motif chemokine ligand 5 (RANTES) were greatly increased in these women, and the PTB-associated bacteria were correlated with proinflammatory cytokines in vaginal fluid. Similarly, Chan et al. (2022) showed that women experienced PTB had depleted vaginal lactobacilli species and high bacterial diversity resulting in increased mannose binding lectin (MBL), IgM, IgG, C3b, C5, IL-8, IL-6, and IL-1β. They also found that preterm labor signals of cervical shortening were associated with depleted *Lactobacillus iners* and elevated levels of IgM, C3b, C5, C5a, and IL-6. These studies linked the vaginal microbiome to PTB, suggesting the potential for vaginal microbes to be early diagnostic biomarkers of PTB risk.

In a case-control study recruiting 94 Korean pregnant women with PTB or term birth, bacteria in the cervicovaginal fluid were used to predict the risk of PTB (Park et al., 2021). It found that PTB risk was low when the ratio of *L. iners* was 0.812 or higher, while in the group with ratios below 0.812, moderate and high risk were classified as a high ratio of *Ureaplasma parvum* (Park et al., 2021). Based on bacterial risk scores, a PTB prediction model created using machine learning (decision tree and support vector machine) had a sensitivity and specificity of 71% and 59%, respectively, whereby vaginal microbes were proposed to predict PTB (Park et al., 2021). However, it has also been reported that using the vaginal microbiome did not always perform well in predicting PTB; instead, machine-learning models (*k-medoids*) based on vaginal metabolites obtained much better accuracy than microbiome (area under receiver operating characteristic curve of 0.78 vs. 0.55) (Kindschuh et al., 2023). Assessment of the contribution of each feature towards the prediction for each sample using SHapley Additive exPlanations (SHAP) captured the microbiome-based predictors including *Mobiluncus mulieris* and *Finegoldia magna*, *Lactobacillus,* and *Dialister* species (Kindschuh et al., 2023). In this regard, the prevalent approach now becomes the integration of multiple data types. The predictive rate can be increased through employing a combination of vaginal microbes, cervical length, and white blood cell count information (Park et al., 2022). Combining vaginal microbiome (features includes *L. crispatus*, *Lactobacillus acidophilus*, etc.), metabolite, maternal host defense molecules from cervicovaginal samples as well as ethnicity information improved the predictive accuracy to an AUC value of 0.835 (Flaviani et al., 2021). It is also intended to perform early prediction of PTB based on personal medical history, clinical characteristics, vaginal microbiome, biophysical characteristics of the cervix, and maternal serum biochemical markers (Becerra-Mojica et al., 2022).

### Auxiliary diagnosis

Multiple studies showed that, except for the gold standards, maternal microbiome has the potential as additional diagnostic proof. Variations of microbiome in different body sites driven by various pregnancy complications were diffusely researched nowadays. The possibility of using maternal microbiome from different body sites as biomarker for GDM has been widely evaluated. Compared to microbiome in stool and vaginal swabs, the oral cavity showed the greatest variation, with more Proteobacteria but fewer Firmicutes found in GDM individuals (Wang et al., 2018), reflecting the feasibility of selecting oral microbes as biomarkers for GDM detection. Accordingly, a GDM classification model based on random forest algorithm was constructed using microbes from saliva and dental plaque to estimate the predictive performance of oral microbiome for GDM (Li et al., 2021). The average area under curve (AUC) value of the classification model reached 0.83 when it was constructed by combination of *Lautropia* and *Neisseria* in dental plaque and *Streptococcus* in saliva (Li et al., 2021). When clinical features were also included, the model performed best with a maximum AUC value of 0.89, indicating that certain bacteria in saliva or dental plaque could distinguish to some extent between women with and without GDM (Li et al., 2021). Notably, *Streptococcus* and *Veillonella* were both depleted in patients with periodontitis and GDM, indicating a reasonable relationship between GDM and periodontitis that related to the decreased abundance of these two genera (Li et al., 2021). Therefore, before getting rid of the interference of periodontitis, the diagnosis of GDM that based on the variations of *Streptococcus* and *Veillonella* should be prudently made.

The development of similar auxiliary diagnostic techniques could also be considered for gestational hypertension and preeclampsia. Pregnant women with gestational hypertension have normal blood pressure (BP) prior to pregnancy but have BP readings of ≥140/90 mmHg on two occasions at least 6 h apart during pregnancy after 20 weeks’ gestation (Sibai, 2003). If there are sustained elevations to 160/110 mmHg for at least 6 h, it should be considered as sever gestational hypertension (ACOG, 2002). Some women with gestational hypertension will subsequently progress to preeclampsia, which is primarily defined as gestational hypertension plus proteinuria (300 mg or more per 24-h period) (Sibai, 2003). Except for the direct phenotype in BP, the abundance of the butyrate-producing genus *Odoribacter* in gut of pregnant women was inversely correlated with systolic BP, indicating that gut microbiome may maintain a normal BP through butyrate (Gomez-Arango et al., 2016). Consistently, significant reductions in short-chain fatty acid-producing bacteria and SCFAs were also observed in preeclamptic patients in late pregnancy (Chang et al., 2020; Jin et al., 2022). *Akkermansia muciniphila*, propionate, or butyrate could significantly moderate the symptoms of preeclamptic rats. A biomarker consortium consisting of *Akkermansia*, *Oscillibacter*, SCFAs in serum and the main clinical indicators (systolic BP, diastolic BP, and urine protein) could reach 98.98% diagnostic efficacy for preeclampsia (Jin et al., 2022). Even without other indexes, the classification model only based on the abundances of *Akkermansia* and *Oscillibacter* still has an accuracy higher than 89%. These results suggested that gut microbiome has great potential for both treatment and diagnosis of preeclampsia.

Existing studies have basically reached a consensus that the decrease of short-chain fatty acid-producing bacteria and SCFAs, especially butyrate, could explain the development of hypertension and preeclampsia of pregnant women to a large extent. The perturbation in the relevant bacteria and metabolites has greatly directive function for the revealing of both therapeutic targets and diagnostic biomarker of gestational hypertension and preeclampsia. However, in future, there still needs a prospective pregnant women cohort to further assess the feasibility of gut microbiome and the metabolites produced by the microbes for the early diagnoses of gestational hypertension and preeclampsia.

### Health monitoring

Compared with early prediction and auxiliary diagnosis, continuous monitoring sometimes is more required. For women at high risk of GDM, early pregnancy screening in the first trimester or at the initiation of antenatal care is generally recommended (IADPSG, 2010; Agarwal et al., 2014; ADA, 2018). However, the diagnosis results of GDM based on OGTT in the first trimester sometimes are opposed to the results in the second or third trimester of pregnancy (Zhu et al., 2013; Johns et al., 2018). Based on a cohort consisting of 17,186 pregnant women, fasting plasma glucose ≥5.1 mmol/L at the first prenatal visit cannot be accepted as the criterion for diagnosis of GDM (Zhu et al., 2013). The reasons of the discordant results between the early and late OGTT are complex that may include detection errors, prolonged fasting, and self-management, etc. But whether the diseases are truly controlled or over-controlled needs longer and more indicative monitoring for GDM before 24 weeks.

In contrast to normoglycemic pregnant women whose gut microbiome has been changing dramatically from the first to the third trimesters (Koren et al., 2012), for pregnant women with GDM, their shift over time was significantly attenuated (Zheng et al., 2020a), suggesting that less dynamic changes in gut microbiome in the first half of pregnancy with GDM. This implies that in addition to the static differences of gut microbiome between GDM and controls, distinct community dynamics between the two groups could also be taken as biomarkers for GDM prolonged monitoring. On the other hand, from the angle of the patients, saliva, or stool sampling are more convenient and acceptable because of the characteristics of noninvasive and non-time-dependent compared with OGTT (Li et al., 2021; Pinto et al., 2023).

Postpartum monitoring of the vaginal microbiome is also essential since the vaginal microbial community shifts significantly from pregnancy to the postpartum period (Zhang et al., 2022). After birth, the *Lactobacillus* genus as well as five of its species (*L. crispatus*, *Lactobacillus gasseri*, *L. iners*, *Lactobacillus jensenii*, and *Lactobacillus reuteri*) were significantly depleted compared with pregnancy, while the nondominant microbes such as *Prevotella*, *Atopobium*, *Acinetobacter*, and *Sneathia* were significantly enriched. This dysbiosis microbiome in vagina was demonstrated to associate with bacterial vaginosis and chronic endometritis in previous studies (Onderdonk et al., 2016; Wang et al., 2021). Postpartum endometritis often occurs when vaginal organisms invade the endometrial cavity during the labor process and cause infection (Mackeen et al., 2015). Chronic endometritis may be a key factor for the lower clinical pregnancy rate (Chen et al., 2021). Hence postnatal vagina microbiome monitoring is very necessary for the early diagnosis of endometritis and promotion of reproductive health.

### Retrospective diagnosis

The microbiome could even be used as a retrospective biomarker of disease in pregnancy (Crusell et al., 2018). The women who suffered GDM had an aberrant microbial composition even about 8 months postpartum compared to normoglycemic pregnant women. In consideration of the FMT results from Pinto et al. (2023) that the gut microbiome in GDM can drive inflammation and insulin resistance, a prolonged microbiome dysbiosis may greatly increase the risk of developing GDM in the next generation. Therefore, a longer term (>2 years) fellow-up of GDM women after delivery is necessary to analyze how long the dysbiosis of gut microbiome caused by GDM persist. These results may be instructive for a woman who suffered GDM in her first born, if she plans to have a second child.

In addition, the postpartum vaginal microbiome showed a strong association with the delivery mode (Zhang et al., 2022). Compared with the pregnant women who delivered vaginally, women who delivered via cesarean section had significantly higher microbial diversity but lower *Lactobacillus* in vagina even 6 weeks after delivery. This phenomenon indicated that the effect of delivery mode on vaginal microbiome is prolonged, the higher microbial diversity and lower abundance of *Lactobacillus* in vagina could be used as a biomarker for retrospective diagnosis for delivery mode. As mentioned above, depleted *Lactobacillus* in vagina was highly associated with bacterial vaginosis and chronic endometritis in previous studies (Onderdonk et al., 2016; Wang et al., 2021). The infection rates for endometritis by type of delivery were: vaginal, 3.6%; elective repeat cesarean section, 6.0%; nonurgent primary cesarean section, 22.2%; and emergency cesarean section, 38.4% (Hawrylyshyn et al., 1981). Retrospective vaginal microbiome analysis of the women who suffered cesarean section may be helpful for the early detection and treatment of endometritis of them. In addition, abortion history and pregnancy complications were also traceable based on the shifts in the relative abundances of certain bacterial species in vagina (Zhang et al., 2022).

It appears that the microbiome has extraordinary potential to predict, diagnose and monitor GDM, gestational hypertension, PTB, and preeclampsia, the most common complications of pregnancy. Compared with the other three pregnant diseases, the researches in the microbiome related with GDM were more sufficient until now and involved in all the four sections we talked about. With wider and wider of the applications of multi-omics including microbiome, utility of microbiome for early detection and monitoring of pregnancy complications, and adverse pregnancy outcomes will continually expand. Due to the high fluctuation and susceptibility of microbiome under various diseases, particularly oral microbiome, early predication of pregnancy complications based on microbiome should be performed with comprehensive consideration of various factors as much as possible.

## Precise management of nutrition and health during pregnancy considering the microbiome

In addition to concern about the incidence of pregnancy complications and adverse pregnancy outcomes, maintaining normal physiological homeostasis and nutritional requirements is key to perinatal care. During pregnancy, a series of physiological changes occurs in various organs of the circulatory, respiratory, digestive, and endocrine system. Nutritional monitoring and dietary interventions that take the microbiome as an indicator or target will bring a conceptual revolution in the precise management of nutrition and health during pregnancy, especially for special populations such as patients with GDM. Prevention of fetal overgrowth and control of maternal hyperglycemia are the primary goal for GDM treatment, which is usually achieved by dietary modification and promotion of physical activity to minimize postprandial glucose elevations (McIntyre et al., 2019), so individualized nutrition and lifestyle guidance are increasingly required for pregnancy management. While advances in digital tools and artificial intelligence can help individuals more easily track nutrient intake, the influence of these nutrients on health outcomes varies widely among individuals, due in part to the heterogeneity of the gut microbiome (Matusheski et al., 2021). Thus, in this section, the associations between microbiome and precise management of nutrition will be discussed. Although these studies were not all specifically conducted on pregnancy and neonates, the results definitely will boost the precise management of nutrition and health during pregnancy.

The heterogeneous community properties of the gut microbiome suggest different metabolic function and responses to the same food in digestive tract, which may eventually influence the maternal and infant metabolism. Two enterotypes of gut microbiome were classified based on 651 Chinese pregnant women, which are separately dominated by *Bacteroides*, *Prevotella* (Yao et al., 2022). Differences in taxonomic composition imply that enterotypes may differ in functional and ecological properties. For example, it has been shown that *Prevotella* enterotype was enriched in individuals with non-Western and/or fiber-rich diets (De Filippo et al., 2010; Smith et al., 2013; David et al., 2014), as *Prevotella*-hydrolases are specialized in the degradation of plant fibers (Purushe et al., 2010). Conversely, *Bacteroides-*enterotype associated with diets enriched in animal protein and saturated fats (David et al., 2014). As expected, recent studies have shown that gut microbiome is closely associated with host glucose metabolism (Zeevi et al., 2015; Sun et al., 2023), although factors that may personally affect postprandial glycemic responses (PPGRs) traditionally also include genetics (Carpenter et al., 2015), lifestyle (Dunstan et al., 2012), insulin sensitivity (Himsworth, 1934), exocrine pancreatic, and glucose transporters activity levels (Gibbs et al., 1995). In this process, microbial functions involved in fiber degradation to short-chain fatty acids (SCFAs) exhibited inverse associations with fasting plasma glucose levels, and the SCFA-producing bacterial enzymes were inversely associated with OGTT results (Sun et al., 2023). This suggested a potential usage of gut microbiome for postprandial glycemic responses predication during pregnancy.

**Figure 2.**
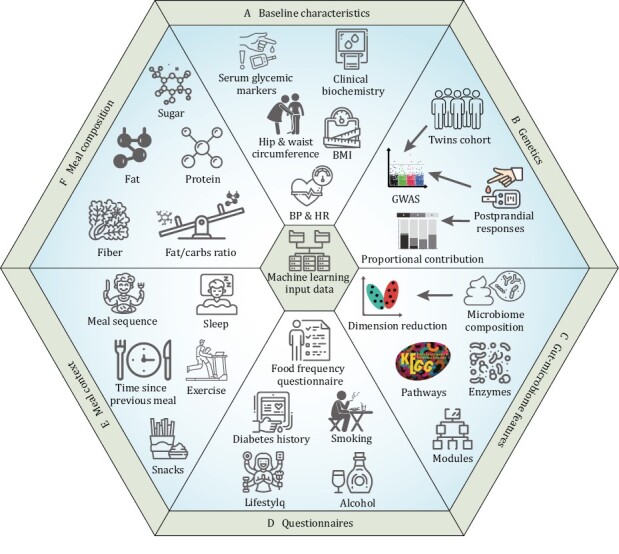
**Features employed in the machine-learning model for the prediction of postprandial glycemic responses.** The features are mainly classified into six groups: (A) baseline characteristics, (B) genetics, (C) gut microbiome features, (D) questionnaires, (E) meal context, and (F) meal composition. Meal context represents the activity or snacks consumption that may influence the glycemic responses after meal. BMI: body mass index; HR: heart rate; Fat/carbs: ratio of fat to carbohydrates content in meals composition; GWAS: genome wide association study.

Indeed, such an approach has been tried in populations. A stochastic gradient boosting regression algorithm that integrates blood parameters, dietary habits, anthropometrics, physical activity, and gut microbiome could accurately predict personalized postprandial glycemic response to real-life meals (Zeevi et al., 2015) (Fig. 2). The algorithm predicted and measured PPGRs with a substantially higher Pearson correlation of 0.7 compared to the reference of carbohydrate and calories counting model that only achieved the performance to 0.38 and 0.33. Another model based on random forest regression using features consisting of meal composition, habitual diet, meal context, anthropometry, genetics, microbiome, clinical, and biochemical parameters has also shown excellent performance in the prediction of glycemic and triglyceride responses to food intake (Berry et al., 2020). In this model, Pearson correlations of the predicted values and the observed values were 0.77 and 0.47 for glycemic and triglyceride responses prediction. Notably, for postprandial lipemia prediction, the contribution of gut microbiome (7.1%) even higher than meal macronutrients data (3.6%) (Berry et al., 2020). These studies raise the possibility of designing personalized dietary interventions for pregnant women with GDM based on the maternal microbiome.

Following this concept, diets with low and high PPGRs predicted by the algorithm were labeled as “good” and “bad” diets, respectively (Zeevi et al., 2015). The experimental results confirmed that PPGRs were significantly higher in the “bad” diet than in the “good” diet, while postprandial glucose fluctuations were significantly lower in the “good” diet than in the “bad” diet. There is no doubt that the microbiome variations from one pregnant woman to the next will be a valuable breakthrough point for precise pregnancy nutrition management. Indeed, a birth cohort [Westlake Precision Birth Cohort (WeBirth)] based on pregnant women with GDM, integrating the utilization of wearable devices including CGM and objective measurements of physical activity, standardized testing meals, daily dietary records, and collection of multi-omics data was established to explore optimal nutrition recommendations for patients with GDM from the perspective of precision nutrition and the association between personalized blood glucose response and birth outcomes (Liang et al., 2023). Further evaluation is still needed for long-term personalized interventions, e.g., for months or even years, the implications are significant. Long-term personalized dietary control of PPGR may help to control, ameliorate, or prevent a range of metabolic disorders of pregnancy associated with long-term impaired glucose control in the prenatal and postnatal periods.

Precise management of nutrition and health during pregnancy is of highly clinical applications value. Due to the highly individual differences across people, precise prediction of post-meal glycemic responses is difficult. To a certain extent, these varied glycemic responses derived from different metabolic ability of gut microbiome. The succeed deployment of prediction model based on the multi-type datasets including gut microbiome made it possible to design personalized diets for pregnant women. But the relevant researches in pregnancy still limited. Combining the personalized data of post-meal glycemic responses and nutrient requirement of pregnancy may further improve the performance of precise management of nutrition and health during pregnancy.

## Intergenerational health care and interventions targeting the maternal microbiome

Maternal microbiome from gut, vagina, mouth, milk, and skin are regarded as the main sources of neonatal microbiota (Wang et al., 2020), with gut strains providing the largest contribution of colonizing microorganisms (Ferretti et al., 2018) and vagina being the primary viruses source of vaginally newborns (Wang et al., 2022). The maternal microbiota changes as a result of having GDM or increasing age, with subsequent synergistic changes in the neonatal microbiota (Wang et al., 2018; Li et al., 2022), which may impact the initial microbiota of infants and affect their long-term health. For example, increased *L. iners* transmission from mothers and neonates was observed under GDM condition (Wang et al., 2018). Due to shortage of genes necessary to synthesize amino acids *de novo* in *L. iners* (Macklaim et al., 2011), a relatively high ratio of this strain may decrease the efficiency of the microbial amino acid metabolism in newborn’s digestive tract. In this section, we discussed utility of maternal microbiome as indicator and target for neonatal health care.

### Intergenerational indicators

In exploring the relationship between age at childbirth and the initial gut microbiota of newborns, we previously found the same trend of microbial changes between mothers and offspring in terms of alpha diversity and Bray-Curtis distance (Li et al., 2022). Similarly, in the case of pregnant women with GDM, we observed a concordance of microbial variations between mothers and neonates, such as *Prevotella*, *Streptococcus,* and *Bacteroides* (Wang et al., 2018). Synergistic microbial variations between mothers and neonates were also observed under autism spectrum disorders (ASDs) (Li et al., 2019). Analysis of beta diversity based on the unweighted UniFrac distances revealed that the microbiome of healthy mothers and children were significantly different from those of ASD mothers and children, while the similarities between mothers and children were relatively high in both healthy and ASD (Li et al., 2019). These observations proved that the maternal microbiome may server as an indicator for neonatal health.

The value of the maternal microbiome as an indicator of offspring health can be presented by the example of intergenerational transmission of microorganisms in relation to neonatal neuro development and the risk of ASD. Maternal obesity during pregnancy was demonstrated that highly associated with increased risk of neurodevelopmental disorders, including ASD, in offspring (Krakowiak et al., 2012; Sullivan et al., 2014; Connolly et al., 2016). Study in mice model showed that maternal high-fat diet (MHFD) induces a shift in microbial ecology that negatively impacts offspring social behavior (Buffington et al., 2016). Metagenomic shotgun sequencing of fecal samples from both MHFD and MRD (mothers on a regular diet) offspring identified several species whose relative abundance was dramatically reduced in the MHFD offspring microbiota with *L. reuteri* was the most drastically reduced in the MHFD microbiota population (Buffington et al., 2016). Treatment with *L. reuteri* rather than other *Lactobacillus* species significantly improved sociability and preference for social novelty in MHFD offspring (Buffington et al., 2016). Similar effects of *L. reuteri* on reversing the deficient social behavior of ASD children/mice were also observed in multiple other studies (Sgritta et al., 2019; Buffington et al., 2021; Yu et al., 2022). Taken together, depleted *L. reuteri* may not only serve as the biomarker or indicator of ASD, but also could be used as a targeted point for this disease therapy. Intriguingly, depleted *L. reuteri* was also observed in the meconium of offspring born from GDM mothers (Wang et al., 2018), suggesting maternal microbiome with GDM during pregnancy may be instructive for newborn’s neurodevelopment after birth. This may explain why GDM onset early in pregnancy may increase ASD risk of the offspring (Jo et al., 2019).

### Intergenerational targets

In addition to the ASD mentioned above, the microbiome can be represented in a broader range as a target for intergenerational interventions. This is because the influences during pregnancy from various exposures are not confined to the mothers, but can spread to neonates and lay impacts on the neonatal immune and health for a prolonged period. For example, mild and transient infections (*Yersinia pseudotuberculosis*) encountered by mammals during timed-pregnancy specially increased the number of TH17 cells in the small and large intestinal lamina propria of her adult offspring through maternal cytokine IL-6 (Lim et al., 2021). Altered epithelial activation status of the offspring could enhance antimicrobial activity to intestinal microbes’ infections (e.g., *Salmonella*). But for cutaneous *Candida albicans* infection there has no significant defense effect, suggesting that maternal imprinting of offspring immunity is restricted to the gut compartment (Lim et al., 2021). Another example is that perinatal exposure to *Helicobacter pylori* extracts or its immunomodulator vacuolating cytotoxin confers robust protective effects against allergic airway inflammation in offspring (Kyburz et al., 2019). The potential mechanism included skewing of regulatory over effector T cells, expansion of regulatory T-cell subsets expressing C-X-C motif chemokine receptor 3 (*CXCR3*) or retinoic acid-related orphan receptor γt, and demethylation of the forkhead box P3 (*FOXP3*) locus (Kyburz et al., 2019). Except for microbial infection, maternal diet may influence the infant gut microbiome through vertical transfer of maternal microbes to infants during vaginal delivery and breastfeeding (Lundgren et al., 2018). Increased fruit intake was associated with an increased odd of belonging to the high *Streptococcus*/*Clostridium* group among infants born vaginally (Lundgren et al., 2018).

Maternal natural antibodies (mNabs) inspired by maternal gut microbiome are also involved in the shaping of offspring immune (Zheng et al., 2020b). Enterotoxigenic *Escherichia coli* (ETEC) colonizes the small intestine of neonatal mice and typically causes acute and lethal diarrhoeal disease. mNab^+^ pups were more resistant to infection than mNab^−^ pups and displayed a 33-fold reduction in intestinal colonization of ETEC (Zheng et al., 2020b). Pups born to germ-free dams immunized with *Pantoea* were significantly more protected against ETEC than pups born to unimmunized germ-free dams (Zheng et al., 2020b). IgG collected from pups born to *Pantoea*-immunized germ-free dams showed cross-reactivity to ETEC and the enteric pathogen *Citrobacter rodentium*, while pups of germ-free unimmunized dams had no detectable antibodies against ETEC (Zheng et al., 2020b). These results suggested that the commensal microbiota can induce antibodies that recognize antigens expressed by enterotoxigenic *E. coli* and other Enterobacteriaceae species, which confers protection against enterotoxigenic *E*. *coli* in pups (Zheng et al., 2020b). Collectively, the above studies implied a potential utility of maternal microbiome as the targeted regulation points that fine-tunes the immune development of the offspring.

In short, microbiome transmission from mothers to neonates is an important factor that is associated with newborn’s disease occurrence. This intergenerational transmission includes the transfer of microbes and microbe-specific antibodies. Synchronous changes between maternal microbiome and neonatal microbiome imply an instructive effect of maternal microbiome for newborn’s health. Diseases or exposures during pregnancy may shape the microbiome and immune development of the offspring, suggesting that maternal microbiome may server as a targeted regulation point for neonatal health care.

## Assessment of infant development and nutritional status based on the microbiome

Compared to maternal health during pregnancy, it appears that not as much attention has been invested in the indicating and targeting role played by the infant microbiome, except in the context of microbiome maturity and malnutrition. However, the importance of early-life microbiome has been acutely recognized. With the advent of a series of large neonatal cohort studies, the resolution of the neonatal microbiome continues to provide new insights into growth status, nutritional levels, and influencing factors in the early years of life. In this section, we will discuss the characteristics of neonatal microbiome and the interactions between gut microbes and malnutrition.

### Indicator of neonatal growth

Neonatal microbiome thrives rapidly after birth. According to the TEDDY study (Stewart et al., 2018), newborn’s gut microbiome developing roughly consists of three distinct phases: a developmental phase (months 3–14), a transitional phase (months 15–30), and a stable phase (months 31–46). Based on the analysis of 13,776 fecal samples or datasets from 1,956 infants between 1 and 3 years of age, we identified a deterministic developmental process of infant gut microbiome (Xiao et al., 2021). As infants grow up, microbiome with unstable community structure and low microbiome maturation gradually turn to that characterized by higher diversity and stronger connections (Huang et al., 2023), namely from stages of immaturity to maturity. During this mature or stable phase, the infants’ gut microbiota was mainly driven by *Bifidobacterium*, *Bacteroides,* and *Prevotella* and the dominated microbes will not change as much as developmental and transitional phase (Xiao et al., 2021). These results implied that maturity of gut microbiome may be a potential indicator of neonatal growth. Accordingly, to characterize gut microbiome maturation, variances of gut microbiome of 12 healthy children were regressed against the chronologic age of each child at the time of fecal sample collection using the random forests machine-learning algorithm (Subramanian et al., 2014). The regression explained 73% of the variance related to chronologic age, and identified 24 most age-discriminatory taxa. Then the trained model was subsequently applied to other healthy children cohort, and found that the model could also be applied (*r*^2^ = 0.71), verifying the utility of microbiome maturity as an indicator of neonatal growth.

**Figure 3.**
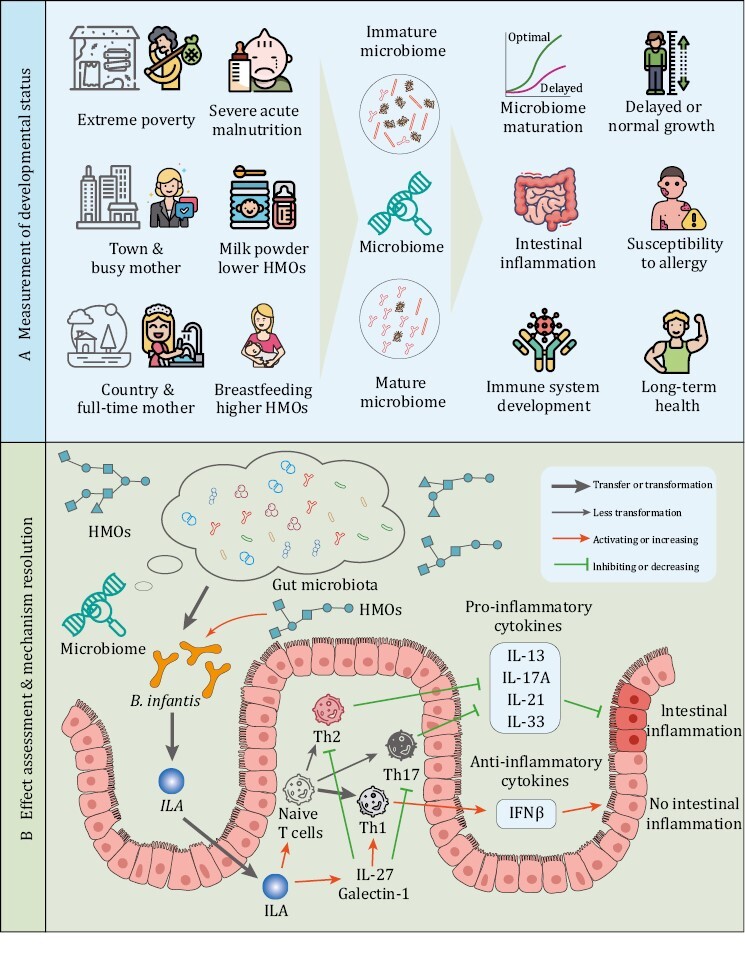
**Measurement of developmental status and effect assessment of infants with microbiome.** (A) The newborns in extreme undeveloped country or town tends to have immature gut microbiome characterized by absent *B*. *infantis* in gut, due to lower HMOs intake. Depleted bifidobacteria is associated with systemic inflammation and immune imbalance early in life. Immature gut microbiome may affect the growth, long-term health, and the susceptibility to allergy. (B) *Bifidobacterium infantis* metabolizes HMOs to indole-3-lactic acid (ILA), which skew naive T cells away from proinflammatory Th17 and Th2 and toward anti-inflammatory Th1. IL-27 limits Th2- and Th17-type responses and regulate T cell function by activating Th1. Galectin-1 induces L-27 and IL-10 and act through IFNβ-dependent reprogramming of tissue macrophages and be essential for inflammation resolving.

Microbiome maturity is also able to measure the impact of a number of factors to which the infant is exposed during growth. The factors include age, geography, delivery mode, feeding mode, preterm, and gestational age (Fig. 3). Thereinto, age and the geographical factor were much stronger than the others (Xiao et al., 2021). Comparative assessment of human fecal microbiota from three age-groups: infants, adults, and the elderly, demonstrated that the human intestinal microbiota undergoes maturation from birth to adulthood and is further altered with aging (Mariat et al., 2009). The microbiota of infants was generally characterized by low levels of total bacteria. *Clostridium leptum* and *Clostridium coccoides* species were highly represented in the microbiota of infants, while elderly subjects exhibited high levels of *E. coli* and *Bacteroidetes* (Mariat et al., 2009). The ratio of *Firmicutes* to *Bacteroidetes* much higher in adults than in infants and elderly (Mariat et al., 2009). For the geographical factor, an important example is *Bifidobacterium longum* subspecies *infantis* (*B*. *infantis*), which dominates the gut microbiomes of young, breastfed children in undeveloped countries [e.g., Bangladesh (Huda et al., 2019) and Malawi (Grześkowiak et al., 2012)], but is nearly absent in children in developed countries [e.g., Europe (Abrahamsson et al., 2014) and North America (Azad et al., 2013)]. *Bifidobacterium infantis* was demonstrated to induce skewing of T cells away from T helper 17 (TH17) and TH2 and toward TH1 cell differentiation through the production of specific metabolites such as indole-3-lactic acid, and exert a dampening effect on systemic and intestinal inflammation (Henrick et al., 2021). These results implied that depleted *B. infantis* in infants’ gut is a potential indicator for higher risk of incidence of immune-mediated diseases such as asthma, allergies, and autoimmune diseases in industrialized societies (Brodin, 2022) (Fig. 3).

Except for gut microbiome, oral microbiota is another possible harbinger for multiple children’s diseases including early childhood caries, celiac diseases, autism, Henoch-schönlein purpura disease, etc. (Xiao et al., 2020). The oral microbiome may also play a vital role and serve as an indicator of infant development. Infant growth was negatively associated with the oral microbial diversity, and positively associated with the Firmicutes-to-Bacteroidetes ratio of the oral microbiota (Craig et al., 2018). *Megasphaera* in oral was demonstrated as a significant mediator between PM_2.5_ exposure and restricted infant growth in the first 3 months after birth (Wu et al., 2022). Although the research related to oral microbiome and infant growth were limited until now compared with gut microbiome, these existed results indicated a similar utility and potential of oral microbiome with gut microbiome as an indicator of neonatal growth.

### Infant and child malnutrition

The infant microbiome is often used as a measure of severe acute malnutrition (SAM), which typically develops between 3 and 24 months after birth (Victora et al., 2010) with weight-for-height Z-scores below three standard deviations (−3 s.d.) from the median of the World Health Organization reference growth standards. SAM was associated with significant relative microbiome immaturity. Persistent immaturity of the gut microbiome existed in children with SAM (Subramanian et al., 2014). Therapeutic food interventions only partially ameliorated anthropometric measurements and microbiome maturity.

A study combining microbiome analysis and germ-free animal studies in a twin cohort provides further evidence that microbiome immaturity causes malnutrition (Smith et al., 2013). Over 300 Malawian twin pairs were treated with ready-to-use therapeutic food (RUTF) or soy-peanut ready-to-use supplementary food (Smith et al., 2013). Among them, 135 twin pairs were discordant in phenotypes restoration. Principal coordinates analysis of Hellinger distances computed from the KEGG enzyme commission number (EC) content of fecal microbiomes demonstrated a different functional development of the gut microbiomes between healthy co-twins and kwashiorkor co-twins (Smith et al., 2013). Further transplantation of fecal microbial communities of discordant twins into gnotobiotic mice induced a distinct response to Malawian and RUTF diets. Kwashiorkor co-twin fecal microbiome recipients became more severely anorectic after 3 weeks Malawian diets, and did not achieve the same body weight as recipients of the healthy sibling’s microbiome after 2 weeks on RUTF (Smith et al., 2013). However, switching from a Malawian diet to RUTF produced a rapid change in configuration of the fecal microbiome with prominent increases in *Bifidobacteria*, *Lactobacilli,* and *Ruminococcus* in kwashiorkor group, indicating that gut microbes may also be regulation target in addition to food-based interventions (Smith et al., 2013). Another study also demonstrated that transplanting immature microbiota from undernourished Malawian children donors into young germ-free mice impaired growth, altered bone morphology, and metabolic abnormalities in the muscle, liver, and brain to recipient gnotobiotic mice (Blanton et al., 2016). Further both cohousing the mice who received healthy or immature microbiome and adding *Ruminococcus gnavus* and *Clostridium symbiosum* to the microbiota from undernourished donors ameliorated growth and metabolic abnormalities in recipient animals (Blanton et al., 2016).

Intriguingly, comparison between the growth parameters of wild-type and germ-free infant male mice showed that germ-free mice weighed 14.5% less and were 4% shorter than wild-type mice even similar amounts of food were ingested relative to body weight (Schwarzer et al., 2016), demonstrating that the gut microbiome contributes to optimal weight gain and longitudinal growth. Further analysis veiled a strain-dependent promotion effect on juvenile growth during chronic undernutrition. A selected lactobacillus strain (*Lactiplantibacillus plantarum*) is sufficient to partly abrogate the growth hormone resistance of peripheral tissues caused by chronic undernutrition. Based on the above results, we imagine that, except for nutritional therapy, targeted microbial interventions may become a novel and complementary strategy to ameliorate the adverse effects of undernutrition on human postnatal growth.

Neonatal microbiome thrives synchronously with newborns growth. As infants grow up, microbiome gradually changes from immaturity to maturity. Nutrition status is a critical influence factor of microbiome maturity. Malnutrition suppresses the development of microbiome. The deterministic and predictable developmental process of infant gut microbiota make it possible to use microbiome as the indicator of neonatal growth and nutritional status.

## Development of maternal and neonatal health products under the guidance of the microbiome

In consideration of the specific physiological stages of pregnancy and infants, although various complications often occur, methods that could be adopted for those diseases’ treatment are limited, particularly for women with chronic diseases and neonates with immune-mediated diseases. In addition to precise nutritional and dietary management mentioned above, over-the-counter probiotics have become an alternative option for the public to improve health during pregnancy as well as in infants and children. Microbiome also plays a critical role in the developments of maternal and neonatal probiotics and prebiotics.

### Discovering probiotics and prebiotics for mothers and infants

Recently, guiding by maternal and infant microbiome studies, probiotic supplementations for pregnant women and neonates were broadly attempted to prevent and regulate the microbiome dysbiosis during pregnancy (Chen et al., 2019; Lopez-Moreno and Aguilera, 2020) as well as for infants during early life, especially for preterm infants (Samara et al., 2022) and cesarean section infants. These infants usually have an abnormal gut microbiome during the first few months, and are mainly deficient in *Lactobacilli* of maternal vaginal origin or *Bifidobacterium*, posing a risk of allergies, asthma, and infections (Yassour et al., 2016; Wampach et al., 2018; Shao et al., 2019). The vast majority of probiotic applications explored in response to these problems are supplemented with such two types of bacteria. For instance, *Lactobacillus* cocktail probiotics (including *L. plantarum* 299v, *Lactobacillus bulgaricus* Lb-87, *Lactobacillus paracasei* DSM 13434, etc.) significantly alleviated the severity of nausea, vomiting, constipation, and improved life quality in pregnancy (Liu et al., 2021), and supplementation with *Lactobacillus rhamnosus* and *Bifidobacterium animalis* reduced the incidence of PTB (Godfrey et al., 2021). *Lactobacillus*, *Bifidobacterium*, *Propionibacterium,* and *Streptococcus*, or a combination of them, bring benefits for the gut microbiome of cesarean-delivered newborns, closing the gap with vaginally delivered neonates, in particular, promoting bifidobacterial colonization (Martín-Peláez et al., 2022). Even more intriguing is the idea of intergenerational supplementation that maternal supplementation of *Lactobacillus johnsonii* relieving airway mucus and TH2 cell-mediated response to respiratory syncytial virus (RSV) infection of offspring mice (Fonseca et al., 2021). Maternal supplementation altered gut microbiome in both mothers and neonates, suggesting that *Lactobacillus* modulation of the maternal microbiome and associated metabolic reprogramming enhanced neonatal airway protection against RSV.

With the guidance of the mother-infant microbiome, reports on probiotic regulation of mother-infant microbiome for health maintenance have become more widespread in the past decade. A typical case is that of HMOs. HMOs are known to be particularly important for the development of the neonatal gut microbiome and immune system (Oozeer et al., 2013; Garrido et al., 2015), as HMOs increase the proportion of *B. longum* subsp. *infantis* (*B*. *infantis*) which has specifically evolved to degrade the complete repertoire of HMOs (Canfora et al., 2015). The main compositions of HMOs consist of 2ʹ-fucosyllactose, 2ʹ,3-di-fucosyllactose, lacto-*N*-tetraose, 3ʹ-sialyllactose, and 6ʹ-sialyllactose (Bosheva et al., 2022). Due to the exorbitant prices of these ingredients, galacto- and fructo-oligosaccharides (GOS and FOS, respectively) are designed to mimic or partially replace HMOs in a ratio 9:1 in infant milk formulas (Oozeer et al., 2013). Similar positive effects of this mixture on stool characteristics such as stool consistency and stool frequency were observed (Scholtens et al., 2014). Most current prebiotics such as galacto-oligosaccharide (GOS), fructo-oligosaccharide (FOS), inulin, and lactulose are carbohydrates, but by definition, prebiotics are not limited to carbohydrates. In mice, administration of curcumin, resveratrol and some polyphenol-rich extracts showed prebiotics effects through increasing the levels of bifidobacteria, lactobacilli, and *Akkermansia* spp. in mice (Bereswill et al., 2010; Neyrinck et al., 2013; Anhê et al., 2015). Maternal and infant microbiome studies are spawning a rapid expansion of probiotic candidate species on the list.

It suggests that the microbiome may assist in the discovery of novel probiotics and prebiotics for mothers and newborns. In addition to the variations in microbial composition, *in silico* analysis of the microbial genomes can also be used to assess whether a probiotic containing colonization-promoting gene functions (Kapse et al., 2019; Alayande et al., 2020), identify interactions between the probiotics and gut symbionts (Gupta et al., 2020), and eventually predict the personalized gut mucosal colonization (Zmora et al., 2018). Further, microbial transcriptomics and proteomics can identify the genes or proteins that are differentially regulated in response to physiological challenges in the gut, which may illuminate *in vivo* adaptation mechanisms of probiotics and the promotion effect of their prebiotics (Johnson et al., 2016; Heavey et al., 2022). Metabolomics can be used to further reveal the ability of the probiotics to apply its metabolic functions to transform host-, microbiota-, and diet-derived and xenobiotic compounds to facilitate survival in the gut (Feng et al., 2020). The metabolites then may affect host health condition (Tang et al., 2023). All these multi-omics results will be determining for the probiotics and prebiotics discovery.

### Safety evaluation and precision application

Although probiotics have achieved enormous popularity among the general public (Clarke et al., 2015; Draper et al., 2017), probiotics efficacy are still discordant at times and remain heterogenous and conflicted among different studies or populations. For instance, probiotics consumption by healthy individuals could alleviate gastrointestinal symptoms (Guyonnet et al., 2009), resist infectious diseases (Panigrahi et al., 2017) and prevent cardio-metabolic disease (Zhang et al., 2015; Sun and Buys, 2016). However, the probiotics efficacy for already existed infections or conditions such as cardio-metabolic or inflammatory bowel diseases remains highly debated (Crovesy et al., 2017; Rondanelli et al., 2017), and even some studies reported probiotics-associated morbidity and mortality (Honeycutt et al., 2007; Besselink et al., 2008; Vogel, 2008). Adverse effects of probiotic consumption may be under-reported in some clinical trials (Bafeta et al., 2018). A comprehensive assessment of probiotic effects is of great necessity to deeply understand high variability in probiotics effects on mothers and infants and their microbiome.

**Figure 4.**
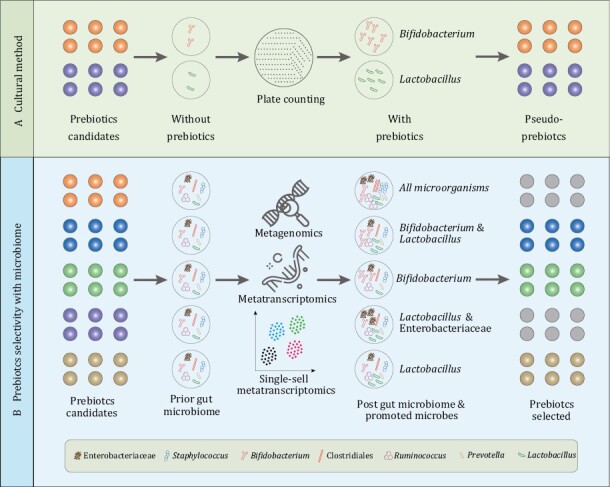
**Differences of the prebiotic selectivity with the methods based on *in vitro* culture and microbiome.** (A) For traditional method, the prebiotics authentication only based on that whether this substrate could promote the proliferation of probiotics such as *Bifidobacterium* and *Lactobacillus*. (B) However, using microbiome, we could comprehensively assess the probiotic effect in real gut microbiome community and screen out the genuine prebiotics.

Likewise, due to the physiological effects of prebiotics, selective utilization of a prebiotic by host microorganisms, metabolic results of this utilization might be the main drivers of the beneficial effect for host health. However, prebiotics occasionally are not the only substances that can affect the microbiome (Savage, 1977). The selective effect of a prebiotics could extend to several microbial groups, but should not all especially the pathogenic or opportunistic microbes. The methods that distinguish prebiotics from many of these other substances will be the basement for the utilization and development of prebiotics. In previous researches based on culture methods, specific stimulation of limited probiotics such as *Lactobacillus* and *Bifidobacterium* was considered a prebiotic effect (Gibson and Roberfroid, 1995). But, because of the complexity of microbial community structure, traditional research methods based on culture *in vitro* cannot comprehensively reveal the impact of prebiotics on other microorganisms except for the targeted probiotics. Prebiotic effects probably extend beyond targeted microbes such as bifidobacteria and lactobacilli in gut ecosystem, but to meet the selectivity criterion of a prebiotic, the range of microorganisms affected must be limited. In other words, the influences of a prebiotics on maternal and infant health should be determined in mixed microbial ecosystems *in vivo* where containing the full microbiome of interest (Fig. 4). The conclusion of prebiotic activity must be based on an assessment of the full microbial diversity, not simply increased abundance of gut bifidobacteria or lactobacilli.

Fortunately, the burgeoning development of high-throughput sequencing has enabled us to identify and measure a broader range of maternal and infant microbial members, which contributes to revealing whether untargeted bacterial genera/species might utilize some prebiotic substrates or be impacted by the prebiotics. Metagenomics and metatranscriptomics allow us to accurately profile the changes of taxonomic composition and functional potential of microbial communities in response to probiotic stimulation (Quince et al., 2017) and to link community genetic potential (gene carriage and abundance) to molecular activity (Bashiardes et al., 2016). In this way, we can comprehensively and objectively assess the impact of probiotics on maternal and infant health, gain insight into the gene expression and functional changes of targeted microbes and improve our understanding of microbial behavior *in situ* under a prebiotic. If the challenges of microbial single-cell transcriptomics could be addressed (Llorens-Rico et al., 2022), combination of metagenomics with single-cell transcriptomics will enormously boost the prebiotic selectivity and assessment of the prebiotic effects, and is expected to lead to the development of more probiotics with promising efficacy for maternal and infant health care.

Overall, the microbiome will have a positive impact on the health maintenance of mothers and newborns. Depleted probiotic strains revealed by microbiome analysis provides a probiotic candidate species list. Further combination of multi-omics data facilitates the probiotics and prebiotics discovery, and promotes the safety evaluation and precision application of them.

## Concluding remarks and future perspectives

Early detection of pregnancy complications and adverse pregnancy outcomes is of great importance for perinatal health care. It requires sensitive and efficient screening indicators, and the microbiome is expected to be one of them (Table 1). Besides static differences in the microbiome, distinct community dynamics could also be taken as biomarkers for health status monitoring during pregnancy. We envision a predictable efficacy for early diagnosis of relevant diseases if we can integrate static and dynamic microbiome data.

**Table 1.** Summary of microbiome as indicators or targets of maternal and neonatal health care.

Objects	Functions	Cases	Microbial indicators or targets	Sources	References
Pregnancy complications and adverse pregnancy outcomes	Early prediction	GDM	*Prevotella copri*↓	Gut	Pinto et al. (2023)
	Early prediction	PTB	*Lactobacillus crispatus*↓ *Lactobacillus iners*↓ *Sneathia amnii*↑ *Prevotella*↑	Vagina	Fettweis et al. (2019)
	Auxiliary diagnosis	GDM	*Lautropia*↑ *Neisseria*↑ *Streptococcus*↓ *Veillonella*↓	Oral	Li et al. (2021)
	Auxiliary diagnosis	Hypertension Preeclampsia	*Odoribacter*↓ *Akkermansia*↓	Gut	Gomez-Arango et al. (2016) Jin et al. (2022)
	Intervention target	Preeclampsia	*Akkermansia muciniphila*↓	Gut	Jin et al. (2022)
	Health monitoring	Endometritis	*Lactobacillus*↓ *Prevotella*↑ *Atopobium*↑ *Acinetobacter*↑ *Sneathia*↑	Vagina	Zhang et al. (2022)
	Retrospective diagnosis	GDM	*Faecalibacterium*↑ *Anaerotruncus*↑ *Clostridium*↓ *Veillonella*↓ *Akkermansia*↓ *Christensenella*↑	Gut	Crusell et al. (2018)
	Retrospective diagnosis	Endometritis	*Lactobacillus*↓	Vagina	Zhang et al. (2022)
	Intervention target	Chronic endometritis	*Lactobacillus murinus*↓	Vagina	Wang et al. (2021)
Precise management of nutrition and health during pregnancy	Precise management	Nutritional status	*Alistipes putredinis*↓ *Eubacterium ramulus*↑ *Bacteroides plebeius*↓	Gut	Sun et al. (2023)
	Precise management	Nutritional status	Proteobacteria↑ Enterobacteriaceae↑ Actinobacteria↑	Gut	Zeevi et al. (2015)
Infant development and nutritional status	Neonatal growth indicator	Developmental status	Firmicutes *Bifidobacterium* *Bacteroides* *Prevotella* *Faecalibacterium rausnitzii* *Ruminococcus* *Lactobacillus ruminis* *Dorea longicatena*	Gut	Xiao et al. (2021) Stewart et al. (2018) Subramanian et al. (2014)
	Diseases indicator	Immune-mediated diseases	*Bifidobacteriumlongum infantis*↓	Gut	Huda et al. (2019) Grześkowiak et al. (2012) Abrahamsson et al. (2014) Azad et al. (2013) Brodin (2022)
	Neonatal growth indicator	Severe acute malnutrition	*Bifidobacteria*↓ *Lactobacilli*↓ *Ruminococcus*↓	Gut	Subramanian et al. (2014)
	Intervention targets	Chronic undernutrition	*Lactobacillus plantarum*↓	Gut	Schwarzer et al. (2016)

The arrows represents the direction of changes (increase:↑ or decrease:↓) of the specific microbes in case (diseases) versus controls.

Although in some cases, microbiome has limited effectiveness in predicting metabolic disorders or dysbiosis in pregnant women, integration of multiple types of data including microbiome, metabolites, and cytokines has achieved desirable performance in early detection of disease in pregnancy. The oral microbiome has great potential for prolonged monitoring of pregnancy health due to its higher convenience and acceptance. But meanwhile it also highly fluctuates than the gut because of food intake and environmental exposure. Therefore, relevant study with oral microbiome should be well controlled to eliminate various inferences. It is important to note that the sensitive bacteria are different for different diseases or different usages, so care needs to be taken in the scope of biomarker use and in excluding interference of different diseases with each other.

Maternal and neonatal microbiome could also assist in the precise management of pregnancy and infants. Machine-learning model based on features consisting of meal composition, habitual diet, meal context, anthropometry, genetics, microbiome, clinical, and biochemical parameters showed an excellent performance in the prediction of glycemic to food intake. Based on the predicated postprandial glycemic responses, we can design better diets with lower fluctuations of postprandial blood glucose for this special population of pregnant women. Long-term personalized dietary control of PPGR may help to control, ameliorate or prevent a range of metabolic disorders of pregnancy associated with long-term impaired glucose control in the prenatal and postnatal periods. In addition to glucose responses, postprandial responses of blood triglyceride and insulin were also predictable based on gut microbiome. On the other hand, considering that maturity of gut microbiome is an indicator of neonatal growth, and that malnutrition induced persistent immaturity of the gut microbiome, targeted microbial interventions may become a novel and complementary strategy to ameliorate the adverse effects of undernutrition on human postnatal growth.

Finally, according to the results of microbiome studies, we note that the use of probiotics or prebiotics in mothers and infants requires extra caution. Both probiotics and prebiotics are maternal and neonatal health products with promising potential. Probiotics efficacy is still discordant at times and remains heterogenous and conflicted among different studies or populations. A comprehensive assessment of probiotic effects is of great necessity to deeply understand high variability in probiotics effects on mothers and infants and their microbiome. Continual progress of multi-omics, especially the microbial single-cell transcriptomics, enable comprehensiveness of person-specific features in tailoring particular/precision probiotics/prebiotics interventions for mothers and infants at various clinical contexts.

## Abbreviations

ASDs: autism spectrum disorders
AUC: area under curve;
auROC: , area under the receiver operating characteristic curve
BP: blood pressure;
CXCL10: C-X-C motif chemokine ligand 10;
CXCR3: C-X-C motif chemokine receptor 3
DOHaD: Developmental Origin of Health and Diseases
EC: KEGG enzyme commission number
ETEC: enterotoxigenic *Escherichia coli*
FMT: fecal microbiota transplant
FOS: fructo-oligosaccharide;
FOXP3: forkhead box P3
GDM: gestational diabetes mellitus
GOS: galacto-oligosaccharide
HMOs: human milk oligosaccharide
IADPSG: International Association of Diabetes and Pregnancy Study Groups
MHFD: maternal high-fat diet;
MIP-1β: , major intrinsic protein of lens fiber-1β
MRD: mothers on a regular diet
OGTT: Oral glucose tolerance test
PPGRs: postprandial glycemic responses
PTB: preterm birth
RANTES: C-C motif chemokine ligand 5
RSV: respiratory syncytial virus
RUTF: ready-to-use therapeutic food
SAM: severe acute malnutrition
SCFAs: short-chain fatty acids
SHAP: SHapley Additive exPlanations
TEDDY: The Environmental Determinants of Type 1 Diabetes in the Young
TH17: T helper 17;
TNF-α: tumor necrosis factor-α
WeBirth: Westlake Precision Birth Cohort

## References

[CIT0001] Abrahamsson TR , Jakobsson HE, Andersson AF et al. Low gut microbiota diversity in early infancy precedes asthma at school age. Clin Exp Allergy 2014;44:842–850. 24330256 10.1111/cea.12253

[CIT0002] ACOG. Diagnosis and management of preeclampsia and eclampsia. Int J Gynecol Obstet 2002;99:159–167. 10.1016/s0029-7844(01)01747-1 16175681

[CIT0003] ADA. Classification and diagnosis of diabetes: standards of medical care in diabetes-2018. Diabetes Care 2018;41:S13–S27. 29222373 10.2337/dc18-S002

[CIT0004] Agarwal M , Boulvain M, Coetzee E et al. Diagnostic criteria and classification of hyperglycaemia first detected in pregnancy: a World Health Organization Guideline. Diabetes Res Clin Pract 2014;103:341–363. 24847517 10.1016/j.diabres.2013.10.012

[CIT0005] Alayande KA , Aiyegoro OA, Nengwekhulu TM et al. Integrated genome-based probiotic relevance and safety evaluation of *Lactobacillus reuteri* PNW1. PLoS One 2020;15:e0235873. 32687505 10.1371/journal.pone.0235873 PMC7371166

[CIT0006] Anhê FF , Roy D, Pilon G et al. A polyphenol-rich cranberry extract protects from diet-induced obesity, insulin resistance and intestinal inflammation in association with increased Akkermansia spp. population in the gut microbiota of mice. Gut 2015;64:872–883. 25080446 10.1136/gutjnl-2014-307142

[CIT0007] Aragón IM , Herrera-Imbroda B, Queipo-Ortuño MI et al. The urinary tract microbiome in health and disease. Eur Urol focus 2018;4:128–138. 28753805 10.1016/j.euf.2016.11.001

[CIT0008] Azad MB , Konya T, Maughan H et al. Gut microbiota of healthy Canadian infants: profiles by mode of delivery and infant diet at 4 months. Can Med Assoc J 2013;185:385–394. 23401405 10.1503/cmaj.121189 PMC3602254

[CIT0009] Bafeta A , Koh M, Riveros C et al. Harms reporting in randomized controlled trials of interventions aimed at modifying microbiota: a systematic review. Ann Intern Med 2018;169:240–247. 30014150 10.7326/M18-0343

[CIT0010] Bashiardes S , Zilberman-Schapira G, Elinav E. Use of metatranscriptomics in microbiome research. Bioinf Biol Insights 2016;10:19–25. 10.4137/BBI.S34610 PMC4839964 27127406

[CIT0011] Becerra-Mojica CH , Parra-Saavedra MA, Diaz-Martinez LA et al. Cohort profile: Colombian Cohort for the Early Prediction of Preterm Birth (COLPRET): early prediction of preterm birth based on personal medical history, clinical characteristics, vaginal microbiome, biophysical characteristics of the cervix and maternal serum biochemical markers. BMJ Open 2022;12:e060556. 10.1136/bmjopen-2021-060556 PMC9152936 35636786

[CIT0012] Beckers KF , Sones JL. Maternal microbiome and the hypertensive disorder of pregnancy, preeclampsia. Am J Physiol Heart Circ Physiol 2020;318:H1–H10. 31626558 10.1152/ajpheart.00469.2019

[CIT0013] Bereswill S , Muñoz M, Fischer A et al. Anti-inflammatory effects of resveratrol, curcumin and simvastatin in acute small intestinal inflammation. PLoS One 2010;5:e15099. 21151942 10.1371/journal.pone.0015099 PMC2997083

[CIT0014] Berry SE , Valdes AM, Drew DA et al. Human postprandial responses to food and potential for precision nutrition. Nat Med 2020;26:964–973. 32528151 10.1038/s41591-020-0934-0 PMC8265154

[CIT0015] Besselink MG , van Santvoort HC, Buskens E et al.; Dutch Acute Pancreatitis Study Group. Probiotic prophylaxis in predicted severe acute pancreatitis: a randomised, double-blind, placebo-controlled trial. Lancet 2008;371:651–659. 18279948 10.1016/S0140-6736(08)60207-X

[CIT0016] Blanton LV , Charbonneau MR, Salih T et al. Gut bacteria that prevent growth impairments transmitted by microbiota from malnourished children. Science 2016;351:add3311–add3317. 10.1126/science.aad3311 PMC4787260 26912898

[CIT0017] Blostein F , Bhaumik D, Davis E et al. Evaluating the ecological hypothesis: early life salivary microbiome assembly predicts dental caries in a longitudinal case-control study. Microbiome 2022;10:240. 36567334 10.1186/s40168-022-01442-5 PMC9791751

[CIT0018] Bosheva M , Tokodi I, Krasnow A et al. Infant formula with a specific blend of five human milk oligosaccharides drives the gut microbiota development and improves gut maturation markers: a randomized controlled trial. Front Nutr 2022;9:1–14. 10.3389/fnut.2022.920362 PMC9298649 35873420

[CIT0019] Brodin P. Immune-microbe interactions early in life: a determinant of health and disease long term. Science 2022;376:945–950. 35617387 10.1126/science.abk2189

[CIT0020] Buffington SA , Di Prisco GV, Auchtung TA et al. Microbial reconstitution reverses maternal diet-induced social and synaptic deficits in offspring. Cell 2016;165:1762–1775. 27315483 10.1016/j.cell.2016.06.001 PMC5102250

[CIT0021] Buffington SA , Dooling SW, Sgritta M et al. Dissecting the contribution of host genetics and the microbiome in complex behaviors. Cell 2021;184:1740–1756.e1716. 33705688 10.1016/j.cell.2021.02.009 PMC8996745

[CIT0022] Canfora EE , Jocken JW, Blaak EE. Short-chain fatty acids in control of body weight and insulin sensitivity. Nat Rev Endocrinol 2015;11:577–591. 26260141 10.1038/nrendo.2015.128

[CIT0023] Carpenter D , Dhar S, Mitchell LM et al. Obesity, starch digestion and amylase: association between copy number variants at human salivary (AMY1) and pancreatic (AMY2) amylase genes. Hum Mol Genet 2015;24:3472–3480. 25788522 10.1093/hmg/ddv098 PMC4498156

[CIT0024] Chan D , Bennett PR, Lee YS et al. Microbial-driven preterm labour involves crosstalk between the innate and adaptive immune response. Nat Commun 2022;13:975. 35190561 10.1038/s41467-022-28620-1 PMC8861006

[CIT0025] Chang Y , Chen Y, Zhou Q et al. Short-chain fatty acids accompanying changes in the gut microbiome contribute to the development of hypertension in patients with preeclampsia. Clin Sci (Colch) 2020;134:289–302. 31961431 10.1042/CS20191253

[CIT0026] Chen Y , Li Z, Tye KD et al. Probiotic supplementation during human pregnancy affects the gut microbiota and immune status. Front Cell Infect Microbiol 2019;9:1–12. 31380297 10.3389/fcimb.2019.00254 PMC6646513

[CIT0027] Chen W , Wei K, He X et al. Identification of uterine microbiota in infertile women receiving in vitro fertilization with and without chronic endometritis. Front Cell Dev Biol 2021;9:1–11. 10.3389/fcell.2021.693267 PMC8409574 34485281

[CIT0028] Cho I , Blaser MJ. The human microbiome: at the interface of health and disease. Nat Rev Genet 2012;13:260–270. 22411464 10.1038/nrg3182 PMC3418802

[CIT0029] Clarke TC , Black LI, Stussman BJ et al. Trends in the use of complementary health approaches among adults: United States, 2002-2012. Nat Health Stat Rep 2015;79:1–16. PMC4573565 25671660

[CIT0030] Connolly N , Anixt J, Manning P et al. Maternal metabolic risk factors for autism spectrum disorder-an analysis of electronic medical records and linked birth data. Autism Res 2016;9:829–837. 26824581 10.1002/aur.1586

[CIT0031] Craig SJC , Blankenberg D, Parodi ACL et al. Child weight gain trajectories linked to oral microbiota composition. Sci Rep 2018;8:14030. 30232389 10.1038/s41598-018-31866-9 PMC6145887

[CIT0032] Crovesy L , Ostrowski M, Ferreira D et al. Effect of Lactobacillus on body weight and body fat in overweight subjects: a systematic review of randomized controlled clinical trials. Int J Obesity 2017;41:1607–1614. 10.1038/ijo.2017.161 28792488

[CIT0033] Crusell MKW , Hansen TH, Nielsen T et al. Gestational diabetes is associated with change in the gut microbiota composition in third trimester of pregnancy and postpartum. Microbiome 2018;6:89. 29764499 10.1186/s40168-018-0472-x PMC5952429

[CIT0034] Damm P , Mathiesen ER. Diabetes: therapy for gestational diabetes mellitus--time for a change? Nat Rev Endocrinol 2015;11:327–328. 25850660 10.1038/nrendo.2015.54

[CIT0035] David LA , Maurice CF, Carmody RN et al. Diet rapidly and reproducibly alters the human gut microbiome. Nature 2014;505:559–563. 24336217 10.1038/nature12820 PMC3957428

[CIT0036] De Filippo C , Cavalieri D, Di Paola M et al. Impact of diet in shaping gut microbiota revealed by a comparative study in children from Europe and rural Africa. Proc Natl Acad Sci USA 2010;107:14691–14696. 20679230 10.1073/pnas.1005963107 PMC2930426

[CIT0037] DiGiulio DB , Romero R, Amogan HP et al. Microbial prevalence, diversity and abundance in amniotic fluid during preterm labor: a molecular and culture-based investigation. PLoS One 2008;3:e3056. 18725970 10.1371/journal.pone.0003056 PMC2516597

[CIT0038] Draper K , Ley C, Parsonnet J. Probiotic guidelines and physician practice: a cross-sectional survey and overview of the literature. Benef Microbes 2017;8:507–519. 28618862 10.3920/BM2016.0146

[CIT0039] Dunstan DW , Kingwell BA, Larsen R et al. Breaking up prolonged sitting reduces postprandial glucose and insulin responses. Diabetes Care 2012;35:976–983. 22374636 10.2337/dc11-1931 PMC3329818

[CIT0040] Fan X , Alekseyenko AV, Wu J et al. Human oral microbiome and prospective risk for pancreatic cancer: a population-based nested case-control study. Gut 2018;67:120–127. 27742762 10.1136/gutjnl-2016-312580 PMC5607064

[CIT0041] Feng L , Raman AS, Hibberd MC et al. Identifying determinants of bacterial fitness in a model of human gut microbial succession. Proc Natl Acad Sci USA 2020;117:2622–2633. 31969452 10.1073/pnas.1918951117 PMC7007522

[CIT0042] Ferretti P , Pasolli E, Tett A et al. Mother-to-infant microbial transmission from different body sites shapes the developing infant gut microbiome. Cell Host Microbe 2018;24:133–145.e135. 30001516 10.1016/j.chom.2018.06.005 PMC6716579

[CIT0043] Fettweis JM , Serrano MG, Brooks JP et al. The vaginal microbiome and preterm birth. Nat Med 2019;25:1012–1021. 31142849 10.1038/s41591-019-0450-2 PMC6750801

[CIT0044] Flaviani F , Hezelgrave NL, Kanno T et al. Cervicovaginal microbiota and metabolome predict preterm birth risk in an ethnically diverse cohort. JCI Insight 2021;6:e149257. 34255744 10.1172/jci.insight.149257 PMC8410012

[CIT0045] Fonseca W , Malinczak CA, Fujimura K et al. Maternal gut microbiome regulates immunity to RSV infection in offspring. J Exp Med 2021;218:e20210235. 34613328 10.1084/jem.20210235 PMC8500238

[CIT0046] Gardella C , Riley DE, Hitti J et al. Identification and sequencing of bacterial rDNAs in culture-negative amniotic fluid from women in premature labor. Am J Perinatol 2004;21:319–323. 15311367 10.1055/s-2004-831884

[CIT0047] Garrido D , Ruiz-Moyano S, Lemay DG et al. Comparative transcriptomics reveals key differences in the response to milk oligosaccharides of infant gut-associated bifidobacteria. Sci Rep 2015;5:13517. 26337101 10.1038/srep13517 PMC4559671

[CIT0048] Gibbs EM , Stock JL, McCoid SC et al. Glycemic improvement in diabetic db/db mice by overexpression of the human insulin-regulatable glucose transporter (GLUT4). J Clin Invest 1995;95:1512–1518. 7706456 10.1172/JCI117823 PMC295634

[CIT0049] Gibson GR , Roberfroid MB. Dietary modulation of the human colonic microbiota: introducing the concept of prebiotics. J Nutr 1995;125:1401–1412. 7782892 10.1093/jn/125.6.1401

[CIT0050] Godfrey KM , Barton SJ, El-Heis S et al.; NiPPeR Study Group. Myo-Inositol, probiotics, and micronutrient supplementation from preconception for glycemia in pregnancy: NiPPeR International Multicenter Double-Blind Randomized Controlled Trial. Diabetes Care 2021;44:1091–1099. 33782086 10.2337/dc20-2515 PMC8132330

[CIT0051] Gomez-Arango LF , Barrett HL, McIntyre HD et al. Increased systolic and diastolic blood pressure is associated with altered gut microbiota composition and butyrate production in early pregnancy. Hypertension 2016;68:974–981. 27528065 10.1161/HYPERTENSIONAHA.116.07910

[CIT0052] Grześkowiak L , Collado MC, Mangani C et al. Distinct gut microbiota in southeastern African and northern European infants. J Pediatr Gastroenterol Nutr 2012;54:812–816. 22228076 10.1097/MPG.0b013e318249039c

[CIT0053] Gupta S , Ross TD, Gomez MM et al. Investigating the dynamics of microbial consortia in spatially structured environments. Nat Commun 2020;11:2418. 32415107 10.1038/s41467-020-16200-0 PMC7228966

[CIT0054] Guyonnet D , Schlumberger A, Mhamdi L et al. Fermented milk containing Bifidobacterium lactis DN-173 010 improves gastrointestinal well-being and digestive symptoms in women reporting minor digestive symptoms: a randomised, double-blind, parallel, controlled study. Br J Nutr 2009;102:1654–1662. 19622191 10.1017/S0007114509990882

[CIT0055] Hanson MA , Gluckman PD. Early developmental conditioning of later health and disease: physiology or pathophysiology? Physiol Rev 2014;94:1027–1076. 25287859 10.1152/physrev.00029.2013 PMC4187033

[CIT0056] Hawrylyshyn PA , Bernstein P, Papsin FR. Risk factors associated with infection following cesarean section. Am J Obstet Gynecol 1981;139:294–298. 7468697 10.1016/0002-9378(81)90013-2

[CIT0057] Heavey MK , Durmusoglu D, Crook N et al. Discovery and delivery strategies for engineered live biotherapeutic products. Trends Biotechnol 2022;40:354–369. 34481657 10.1016/j.tibtech.2021.08.002 PMC8831446

[CIT0058] Henrick BM , Rodriguez L, Lakshmikanth T et al. Bifidobacteria-mediated immune system imprinting early in life. Cell 2021;184:3884–3898.e11. 34143954 10.1016/j.cell.2021.05.030

[CIT0059] Hillier SL , Martius J, Krohn M et al. A case-control study of chorioamnionic infection and histologic chorioamnionitis in prematurity. N Engl J Med 1988;319:972–978. 3262199 10.1056/NEJM198810133191503

[CIT0060] Hillier SL , Witkin SS, Krohn MA et al. The relationship of amniotic fluid cytokines and preterm delivery, amniotic fluid infection, histologic chorioamnionitis, and chorioamnion infection. Obstet Gynecol 1993;81:941–948. 8497360

[CIT0061] Himsworth HP. Dietetic factors influencing the glucose tolerance and the activity of insulin. J Physiol 1934;81:29–48. 16994524 10.1113/jphysiol.1934.sp003113 PMC1394223

[CIT0062] Hoffman DJ , Powell TL, Barrett ES et al. Developmental origins of metabolic diseases. Physiol Rev 2021;101:739–795. 33270534 10.1152/physrev.00002.2020 PMC8526339

[CIT0063] Honeycutt TC , El Khashab M, Wardrop RM et al. Probiotic administration and the incidence of nosocomial infection in pediatric intensive care: a randomized placebo-controlled trial. Pediatr Crit Care Med 2007;8:452–458. 17693918 10.1097/01.PCC.0000282176.41134.E6

[CIT0064] Huang L , Cai M, Li L et al. Gut microbiota changes in preeclampsia, abnormal placental growth and healthy pregnant women. BMC Microbiol 2021;21:265. 34607559 10.1186/s12866-021-02327-7 PMC8489045

[CIT0065] Huang L , Pan G, Feng Y et al. Microbial network signatures of early colonizers in infants with eczema. iMeta 2023;2:1–16. 10.1002/imt2.90 PMC10989766 38868421

[CIT0066] Huda MN , Ahmad SM, Alam MJ et al. Bifidobacterium abundance in early infancy and vaccine response at 2 years of age. Pediatrics 2019;143:e20181489. 30674610 10.1542/peds.2018-1489 PMC6361348

[CIT0067] IADPSG. International association of diabetes and pregnancy study groups recommendations on the diagnosis and classification of hyperglycemia in pregnancy. Diabetes Care 2010;33:676–682. 20190296 10.2337/dc09-1848 PMC2827530

[CIT0068] Jin J , Gao L, Zou X et al. Gut dysbiosis promotes preeclampsia by regulating macrophages and trophoblasts. Circul Res 2022;131:492–506. 10.1161/CIRCRESAHA.122.320771 35950704

[CIT0069] Jo H , Eckel SP, Chen J-C et al. Gestational diabetes mellitus, prenatal air pollution exposure, and autism spectrum disorder. Environ Int 2019;133:105110. 31610366 10.1016/j.envint.2019.105110 PMC7250244

[CIT0070] Johns EC , Denison FC, Norman JE et al. Gestational diabetes mellitus: mechanisms, treatment, and complications. Trends Endocrinol Metab 2018;29:743–754. 30297319 10.1016/j.tem.2018.09.004

[CIT0071] Johnson BR , Hymes J, Sanozky-Dawes R et al. Conserved S-layer-associated proteins revealed by exoproteomic survey of S-layer-forming Lactobacilli. Appl Environ Microbiol 2016;82:134–145. 26475115 10.1128/AEM.01968-15 PMC4702614

[CIT0072] Juan J , Yang H. Prevalence, prevention, and lifestyle intervention of gestational diabetes mellitus in China. Int J Env Res Public Health 2020;17:1–14. 10.3390/ijerph17249517 PMC7766930 33353136

[CIT0073] Kapse NG , Engineer AS, Gowdaman V et al. Functional annotation of the genome unravels probiotic potential of Bacillus coagulans HS243. Genomics 2019;111:921–929. 29859262 10.1016/j.ygeno.2018.05.022

[CIT0074] Kindschuh WF , Baldini F, Liu MC et al. Preterm birth is associated with xenobiotics and predicted by the vaginal metabolome. Nat Microbiol 2023;8:246–259. 36635575 10.1038/s41564-022-01293-8 PMC9894755

[CIT0075] Koren O , Goodrich JK, Cullender TC et al. Host remodeling of the gut microbiome and metabolic changes during pregnancy. Cell 2012;150:470–480. 22863002 10.1016/j.cell.2012.07.008 PMC3505857

[CIT0076] Krakowiak P , Walker CK, Bremer AA et al. Maternal metabolic conditions and risk for autism and other neurodevelopmental disorders. Pediatrics 2012;129:e1121–e1128. 22492772 10.1542/peds.2011-2583 PMC3340592

[CIT0077] Kyburz A , Fallegger A, Zhang X et al. Transmaternal *Helicobacter pylori* exposure reduces allergic airway inflammation in offspring through regulatory T cells. J Allergy Clin Immunol 2019;143:1496–1512.e1411. 30240703 10.1016/j.jaci.2018.07.046 PMC6592617

[CIT0078] Lende M , Rijhsinghani A. Gestational diabetes: overview with emphasis on medical management. Int J Env Res Public Health 2020;17:1–12. 10.3390/ijerph17249573 PMC7767324 33371325

[CIT0079] Leung H , Long X, Ni Y et al. Risk assessment with gut microbiome and metabolite markers in NAFLD development. Sci Transl Med 2022;14:eabk0855. 35675435 10.1126/scitranslmed.abk0855 PMC9746350

[CIT0080] Li N , Yang J, Zhang J et al. Correlation of gut microbiome between ASD children and mothers and potential biomarkers for risk assessment. Genomics Proteomics Bioinformatics 2019;17:26–38. 31026579 10.1016/j.gpb.2019.01.002 PMC6520911

[CIT0081] Li N , An H, Li Z et al. Preconception blood pressure and risk of gestational hypertension and preeclampsia: a large cohort study in China. Hypertens Res 2020;43:956–962. 32322045 10.1038/s41440-020-0438-9

[CIT0082] Li X , Zheng J, Ma X et al. The oral microbiome of pregnant women facilitates gestational diabetes discrimination. J Genet Genomics 2021;48:32–39. 33663937 10.1016/j.jgg.2020.11.006

[CIT0083] Li X , Zhang B, Zheng J et al. Clinical biochemical indicators and intestinal microbiota testing reveal the influence of reproductive age extending from the mother to the offspring. Microbiol Spectr 2022;10:e01076–e01022. 35993782 10.1128/spectrum.01076-22 PMC9602618

[CIT0084] Liang X , Miao Z, Lu S et al. Integration of multiomics with precision nutrition for gestational diabetes: study protocol for the Westlake Precision Birth Cohort. iMeta 2023;2:1–12.

[CIT0085] Lim AI , McFadden T, Link VM et al. Prenatal maternal infection promotes tissue-specific immunity and inflammation in offspring. Science 2021;373:1–14. 10.1126/science.abf3002 34446580

[CIT0086] Liu AT , Chen S, Jena PK et al. Probiotics improve gastrointestinal function and life quality in pregnancy. Nutrients 2021;13:3931. 34836186 10.3390/nu13113931 PMC8624890

[CIT0087] Llorens-Rico V , Simcock JA, Huys GRB et al. Single-cell approaches in human microbiome research. Cell 2022;185:2725–2738. 35868276 10.1016/j.cell.2022.06.040

[CIT0088] Lopez-Moreno A , Aguilera M. Probiotics dietary supplementation for modulating endocrine and fertility microbiota dysbiosis. Nutrients 2020;12:757. 32182980 10.3390/nu12030757 PMC7146451

[CIT0089] Lundgren SN , Madan JC, Emond JA et al. Maternal diet during pregnancy is related with the infant stool microbiome in a delivery mode-dependent manner. Microbiome 2018;6:109. 29973274 10.1186/s40168-018-0490-8 PMC6033232

[CIT0090] Mackeen AD , Packard RE, Ota E et al. Antibiotic regimens for postpartum endometritis. The Cochrane Database Syst Rev 2015;2015:1–99. 10.1002/14651858.CD001067.pub3 PMC7050613 25922861

[CIT0091] Macklaim JM , Gloor GB, Anukam KC et al. At the crossroads of vaginal health and disease, the genome sequence of *Lactobacillus iners* AB-1. Proc Natl Acad Sci USA 2011;108:4688–4695. 21059957 10.1073/pnas.1000086107 PMC3063587

[CIT0092] Mariat D , Firmesse O, Levenez F et al. The Firmicutes/Bacteroidetes ratio of the human microbiota changes with age. BMC Microbiol 2009;9:123. 19508720 10.1186/1471-2180-9-123 PMC2702274

[CIT0093] Martín-Peláez S , Cano-Ibáñez N, Pinto-Gallardo M et al. The impact of probiotics, prebiotics, and synbiotics during pregnancy or lactation on the intestinal microbiota of children born by cesarean section: a systematic review. Nutrients 2022;14:341. 35057522 10.3390/nu14020341 PMC8778982

[CIT0094] Matusheski NV , Caffrey A, Christensen L et al. Diets, nutrients, genes and the microbiome: recent advances in personalised nutrition. Br J Nutr 2021;126:1489–1497. 33509307 10.1017/S0007114521000374 PMC8524424

[CIT0095] McIntyre HD , Catalano P, Zhang C et al. Gestational diabetes mellitus. Nat Rev Dis Primers 2019;5:47. 31296866 10.1038/s41572-019-0098-8

[CIT0096] Neyrinck AM , Van Hée VF, Bindels LB et al. Polyphenol-rich extract of pomegranate peel alleviates tissue inflammation and hypercholesterolaemia in high-fat diet-induced obese mice: potential implication of the gut microbiota. Br J Nutr 2013;109:802–809. 22676910 10.1017/S0007114512002206

[CIT0097] Olabi B , Bhopal R. Diagnosis of diabetes using the oral glucose tolerance test. Bmj 2009;339:b4354. 19864355 10.1136/bmj.b4354

[CIT0098] Onderdonk AB , Delaney ML, Fichorova RN. The human microbiome during bacterial vaginosis. Clin Microbiol Rev 2016;29:223–238. 26864580 10.1128/CMR.00075-15 PMC4786887

[CIT0099] Oozeer R , van Limpt K, Ludwig T et al. Intestinal microbiology in early life: specific prebiotics can have similar functionalities as human-milk oligosaccharides. Am J Clin Nutr 2013;98:561S561s–561S571S. 10.3945/ajcn.112.038893 23824728

[CIT0100] Panigrahi P , Parida S, Nanda NC et al. A randomized synbiotic trial to prevent sepsis among infants in rural India. Nature 2017;548:407–412. 28813414 10.1038/nature23480

[CIT0101] Park S , Oh D, Heo H et al. Prediction of preterm birth based on machine learning using bacterial risk score in cervicovaginal fluid. Am J Reprod Immunol 2021;86:e13435. 33905152 10.1111/aji.13435

[CIT0102] Park S , Moon J, Kang N et al. Predicting preterm birth through vaginal microbiota, cervical length, and WBC using a machine learning model. Front Microbiol 2022;13:1–11. 10.3389/fmicb.2022.912853 PMC9378785 35983325

[CIT0147] Plows JF , Stanley JL, Baker PN et al. The Pathophysiology of Gestational Diabetes Mellitus. Int J Mol Sci 2018;19:3342. 30373146 10.3390/ijms19113342 PMC6274679

[CIT0103] Pinto Y , Frishman S, Turjeman S et al. Gestational diabetes is driven by microbiota-induced inflammation months before diagnosis. Gut 2023;0:1–11. 10.1136/gutjnl-2022-328406 PMC10086485 36627187

[CIT0104] Purushe J , Fouts DE, Morrison M et al. Comparative genome analysis of *Prevotella ruminicola* and *Prevotella bryantii*: insights into their environmental niche. Microb Ecol 2010;60:721–729. 20585943 10.1007/s00248-010-9692-8

[CIT0105] Quince C , Walker AW, Simpson JT et al. Shotgun metagenomics, from sampling to analysis. Nat Biotechnol 2017;35:833–844. 28898207 10.1038/nbt.3935

[CIT0106] Romero R , Sirtori M, Oyarzun E et al. Infection and labor. V. Prevalence, microbiology, and clinical significance of intraamniotic infection in women with preterm labor and intact membranes. Am J Obstet Gynecol 1989;161:817–824. 2675611 10.1016/0002-9378(89)90409-2

[CIT0107] Rondanelli M , Faliva MA, Perna S et al. Using probiotics in clinical practice: where are we now? A review of existing meta-analyses. Gut Microbes 2017;8:521–543. 28640662 10.1080/19490976.2017.1345414 PMC5730384

[CIT0108] Samara J , Moossavi S, Alshaikh B et al. Supplementation with a probiotic mixture accelerates gut microbiome maturation and reduces intestinal inflammation in extremely preterm infants. Cell Host Microbe 2022;30:696–711.e5. 35550672 10.1016/j.chom.2022.04.005

[CIT0109] Savage DC. Microbial ecology of the gastrointestinal tract. Annu Rev Microbiol 1977;31:107–133. 334036 10.1146/annurev.mi.31.100177.000543

[CIT0110] Scholtens PA , Goossens DAM, Staiano A. Stool characteristics of infants receiving short-chain galacto-oligosaccharides and long-chain fructo-oligosaccharides: a review. World J Gastroenterol 2014;20:13446–13452. 25309075 10.3748/wjg.v20.i37.13446 PMC4188896

[CIT0111] Schwarzer M , Makki K, Storelli G et al. Lactobacillus plantarum strain maintains growth of infant mice during chronic undernutrition. Science 2016;351:854–857. 26912894 10.1126/science.aad8588

[CIT0112] Sgritta M , Dooling SW, Buffington SA et al. Mechanisms underlying microbial-mediated changes in social behavior in mouse models of autism spectrum disorder. Neuron 2019;101:246–259.e246. 30522820 10.1016/j.neuron.2018.11.018 PMC6645363

[CIT0113] Shao Y , Forster SC, Tsaliki E et al. Stunted microbiota and opportunistic pathogen colonization in caesarean-section birth. Nature 2019;574:117–121. 31534227 10.1038/s41586-019-1560-1 PMC6894937

[CIT0114] Sibai BM. Diagnosis and management of gestational hypertension and preeclampsia. Obstet Gynecol 2003;102:181–192. 12850627 10.1016/s0029-7844(03)00475-7

[CIT0115] Smith MI , Yatsunenko T, Manary MJ et al. Gut microbiomes of Malawian twin pairs discordant for kwashiorkor. Science 2013;339:548–554. 23363771 10.1126/science.1229000 PMC3667500

[CIT0116] Stewart CJ , Ajami NJ, O’Brien JL et al. Temporal development of the gut microbiome in early childhood from the TEDDY study. Nature 2018;562:583–588. 30356187 10.1038/s41586-018-0617-x PMC6415775

[CIT0117] Subramanian S , Huq S, Yatsunenko T et al. Persistent gut microbiota immaturity in malnourished Bangladeshi children. Nature 2014;510:417–421. 24896187 10.1038/nature13421 PMC4189846

[CIT0118] Sullivan EL , Nousen EK, Chamlou KA. Maternal high fat diet consumption during the perinatal period programs offspring behavior. Physiol Behav 2014;123:236–242. 23085399 10.1016/j.physbeh.2012.07.014 PMC3594403

[CIT0119] Sun J , Buys NJ. Glucose- and glycaemic factor-lowering effects of probiotics on diabetes: a meta-analysis of randomised placebo-controlled trials. Br J Nutr 2016;115:1167–1177. 26899960 10.1017/S0007114516000076

[CIT0120] Sun Z , Pan XF, Li X et al. The gut microbiome dynamically associates with host glucose metabolism throughout pregnancy: longitudinal findings from a matched case-control study of gestational diabetes mellitus. Adv Sci 2023;22:e2205289. 10.1002/advs.202205289 PMC10074094 36683149

[CIT0121] Tang B , Tang L, Li S et al. Gut microbiota alters host bile acid metabolism to contribute to intrahepatic cholestasis of pregnancy. Nat Commun 2023;14:1305. 36894566 10.1038/s41467-023-36981-4 PMC9998625

[CIT0122] Teng F , Yang F, Huang S et al. Prediction of early childhood caries via spatial-temporal variations of oral microbiota. Cell Host Microbe 2015;18:296–306. 26355216 10.1016/j.chom.2015.08.005

[CIT0123] Victora CG , de Onis M, Hallal PC et al. Worldwide timing of growth faltering: revisiting implications for interventions. Pediatrics 2010;125:e473–e480. 20156903 10.1542/peds.2009-1519

[CIT0124] Vogel G. Deaths prompt a review of experimental probiotic therapy. Science 2008;319:557–557. 18239097 10.1126/science.319.5863.557a

[CIT0125] Wampach L , Heintz-Buschart A, Fritz JV et al. Birth mode is associated with earliest strain-conferred gut microbiome functions and immunostimulatory potential. Nat Commun 2018;9:5091. 30504906 10.1038/s41467-018-07631-x PMC6269548

[CIT0126] Wang J , Zheng J, Shi W et al. Dysbiosis of maternal and neonatal microbiota associated with gestational diabetes mellitus. Gut 2018;67:1614–1625. 29760169 10.1136/gutjnl-2018-315988 PMC6109274

[CIT0127] Wang S , Ryan CA, Boyaval P et al. Maternal vertical transmission affecting early-life microbiota development. Trends Microbiol 2020;28:28–45. 31492538 10.1016/j.tim.2019.07.010

[CIT0128] Wang J , Li Z, Ma X et al. Translocation of vaginal microbiota is involved in impairment and protection of uterine health. Nat Commun 2021;12:4191. 34234149 10.1038/s41467-021-24516-8 PMC8263591

[CIT0129] Wang J , Xiao L, Xiao B et al. Maternal and neonatal viromes indicate the risk of offspring’s gastrointestinal tract exposure to pathogenic viruses of vaginal origin during delivery. mLife 2022;1:303–310. 10.1002/mlf2.12034 PMC10989755 38818221

[CIT0130] Weinert LS. International Association of Diabetes and Pregnancy Study Groups recommendations on the diagnosis and classification of hyperglycemia in pregnancy: comment to the International Association of Diabetes and Pregnancy Study Groups Consensus Panel. Diabetes Care 2010;33:e97–e97. 20190296 10.2337/dc09-1848 PMC2827530

[CIT0131] Whiteside SA , Razvi H, Dave S et al. The microbiome of the urinary tract--a role beyond infection. Nat Rev Urol 2015;12:81–90. 25600098 10.1038/nrurol.2014.361

[CIT0132] Wu H , Dong C, Xiao W et al. Associations between PM(2.5) exposure and infant growth: a mediation analysis of oral microbiota. Sci Total Environ 2022;823:153688. 35131243 10.1016/j.scitotenv.2022.153688

[CIT0133] Xiao J , Fiscella KA, Gill SR. Oral microbiome: possible harbinger for children’s health. Int J Oral Sci 2020;12:12. 32350240 10.1038/s41368-020-0082-x PMC7190716

[CIT0134] Xiao L , Wang J, Zheng J et al. Deterministic transition of enterotypes shapes the infant gut microbiome at an early age. Genome Biol 2021;22:1–21. 34429130 10.1186/s13059-021-02463-3 PMC8383385

[CIT0135] Yang Y , Le Ray I, Zhu J et al. Preeclampsia prevalence, risk factors, and pregnancy outcomes in Sweden and China. JAMA Netw Open 2021;4:e218401. 33970258 10.1001/jamanetworkopen.2021.8401 PMC8111481

[CIT0136] Yao X , Zuo N, Guan W et al. Association of gut microbiota enterotypes with blood trace elements in women with infertility. Nutrients 2022;14:3195. 35956371 10.3390/nu14153195 PMC9370633

[CIT0137] Yassour M , Vatanen T, Siljander H et al. Natural history of the infant gut microbiome and impact of antibiotic treatment on bacterial strain diversity and stability. Sci Transl Med 2016;8:1–12. 10.1126/scitranslmed.aad0917 PMC5032909 27306663

[CIT0138] Yoon BH , Romero R, Moon JB et al. Clinical significance of intra-amniotic inflammation in patients with preterm labor and intact membranes. Am J Obstet Gynecol 2001;185:1130–1136. 11717646 10.1067/mob.2001.117680

[CIT0139] Yu Y , Zhang B, Ji P et al. Changes to gut amino acid transporters and microbiome associated with increased E/I ratio in Chd8(+/-) mouse model of ASD-like behavior. Nat Commun 2022;13:1151. 35241668 10.1038/s41467-022-28746-2 PMC8894489

[CIT0140] Zeevi D , Korem T, Zmora N et al. Personalized nutrition by prediction of glycemic responses. Cell 2015;163:1079–1094. 26590418 10.1016/j.cell.2015.11.001

[CIT0141] Zhang Q , Wu Y, Fei X. Effect of probiotics on body weight and body-mass index: a systematic review and meta-analysis of randomized, controlled trials. Int J Food Sci Nutr 2015;67:571–580. 27149163 10.1080/09637486.2016.1181156

[CIT0142] Zhang X , Zhai Q, Wang J et al. Variation of the vaginal microbiome during and after pregnancy in Chinese women. Genomics Proteomics Bioinformatics 2022;20:322–333. 35093602 10.1016/j.gpb.2021.08.013 PMC9684158

[CIT0143] Zheng W , Xu Q, Huang W et al. Gestational diabetes mellitus is associated with reduced dynamics of gut microbiota during the first half of pregnancy. mSystems2020a;5:e00109–e00120. 32209715 10.1128/mSystems.00109-20 PMC7093821

[CIT0144] Zheng W , Zhao W, Wu M et al. Microbiota-targeted maternal antibodies protect neonates from enteric infection. Nature2020b;577:543–548. 31915378 10.1038/s41586-019-1898-4 PMC7362890

[CIT0145] Zhu WW , Yang HX, Wei YM et al. Evaluation of the value of fasting plasma glucose in the first prenatal visit to diagnose gestational diabetes mellitus in china. Diabetes Care 2013;36:586–590. 23193214 10.2337/dc12-1157 PMC3579369

[CIT0146] Zmora N , Zilberman-Schapira G, Suez J et al. Personalized gut mucosal colonization resistance to empiric probiotics is associated with unique host and microbiome features. Cell 2018;174:1388–1405.e1321. 30193112 10.1016/j.cell.2018.08.041

